# Evaluation of the breast cancer care network within the Lazio Region (Central Italy)

**DOI:** 10.1371/journal.pone.0238562

**Published:** 2020-09-03

**Authors:** Margherita Ferranti, Luigi Pinnarelli, Alessandro Rosa, Roberta Pastorino, Mariangela D’Ovidio, Danilo Fusco, Marina Davoli

**Affiliations:** 1 Department of Epidemiology of Lazio Regional Health Service, Rome, Italy; 2 Department of Woman and Child Health and Public Health—Public Health Area, Fondazione Policlinico Universitario A. Gemelli IRCCS, Roma, Italy; 3 Lazio Regional Health Service, Department of Health Information Systems, Rome, Italy; Sapienza University of Rome, ITALY

## Abstract

**Background and objectives:**

A summary indicator for evaluating the breast cancer network has never been measured at the regional level. The aim is to design treemaps providing a summary description of hospitals (including breast units) and Local Health Units (LHUs) in terms of their levels of performance within the breast cancer network of the Lazio region (central Italy). The treemap structure has an intuitive design and displays information from both general and specific analyses.

**Methods:**

Patients admitted to the regional hospitals for malignant breast cancer (MBC) surgery in 2010–2017 were selected in a population-based cohort study.

These quality indicators were calculated based on the international guidelines (EUSOMA, ESMO) to assess the performance in terms of volume of activity, surgery procedure, post-surgery assistance and timeliness of medical therapy or radiotherapy beginning. The quality indicators were calculated using administrative health data systematically collected at the regional level and were included in the treemap to represent the surgery or the post-surgery areas of the breast cancer clinical pathway.

In order to allow aggregation of scores for different indicators belonging to the same clinical area, up to five evaluation classes were defined using the "Jenks Natural Breaks" algorithm. A score and a colour were assigned to each clinical area based on the ranking of the indicators involved. The analyses were performed on an annual basis, by the LHU of residence and by the hospital which performed the surgical intervention.

**Results:**

In 2017, 6218 surgical interventions for MBC were performed in the hospitals of Lazio. The results showed a continuous increase of the level of performance over the years. Hospitals showed higher variability in the levels of performance than the LHUs. 36% of the evaluated hospitals reached a high level of performance. An audit of the S. Filippo Neri breast unit revealed incorrect coding of the input data. For this reason, the score for the indicator for the volume of wards was re-calculated and re-evaluated, with a subsequent improvement of the level of performance. Most LHUs achieved at least an average overall level of performance, with 20% of the LHUs reaching a high level of performance.

**Conclusions:**

This is the first attempt to apply the treemap logic to a single clinical network, in order to obtain a summary indicator for the evaluation of the breast cancer care network. Our results supply decision makers with a transparent instrument of governance for heterogeneous users, directing efforts improving and promoting equity of care. The treemaps could be reproduced and adapted for other local contexts, in order to limit inappropriateness and ensure uniform levels of breast cancer care within local areas. The next step is the evaluation of audit and feedback interventions to improve the quality of care and to guarantee homogeneous levels of care throughout the region.

## Introduction

Breast cancer accounts for 29% of female neoplasms in Italy [1]; one woman in eight is diagnosed with breast cancer. It is the most common cancer in all age groups, although the percentages differ (41% for 0–49 years, 35% for 50–69 years, 22% for 70+ years). It is also the main cause of cancer-related deaths among women (17%) [2]. The incidence of breast cancer appears to be stable, while mortality continues to decline significantly (-0.9% per year).

The organizational model for breast cancer management in Italy focuses on the development of an integrated territory-hospital network, which is organized according to specific diagnosis and treatment services [3], and the institution of breast units. This is based on evidence that the treatment of breast cancer by specialized, multidisciplinary structures with a high volume of activity, guarantees the best outcome in terms of survival and quality of life, and optimizes resources. The breast unit is identified as a hospital with oncology beds and inpatient oncology activities, able to perform magnetic resonance imaging and radiotherapy. To guarantee homogeneous levels of assistance throughout the country [4], hospitals integrate their functions with other local services, through the standardized adoption of European and national guidelines (EUSOMA [5], ESMO [6], AIOM [7,8]). Local Health Units (LHU) have the task of ensuring that networks within their local areas are efficient, managing the overall services provided to their residents with a commitment to continuous improvement [3,4].

The decree of the specially-appointed commissioner (DCA 38/2015) [9] of the Lazio region (central Italy) established this clinical network, in order to ensure the care of patients with a diagnosis of breast cancer. The decree identified 15 hospitals in Lazio as breast units: most of these are located in the Rome metropolitan area. The LHUs outside of the province of Rome each have one breast unit.

Despite this, the 2016 regional welfare organization model was not compliant with the aforementioned decree, and presented significant critical issues: fragmented and uncoordinated care activity; a lack of integration between hospital and local structures, numerous breast units below the surgery volume required, with non-specialized personnel, and insufficient multidisciplinary approaches to breast cancer treatment [10].

The available scientific evidence on quality of care for breast cancer shows an association between patterns of care and outcome [11–13]_._ Assessment of the quality of care using administrative information systems is an opportunity for healthcare improvement. The whole network is accurately evaluated through data systems which allow the monitoring of process and outcome indicators [9]. The use of quality indicators for breast cancer is a tool for improving the performance level of hospitals and LHUs [8]. They can be used to highlight potential problems, areas which need further investigation through clinical studies and research, and to map changes over time.

For this reason, the Lazio Regional Outcome Evaluation Program (P.Re.Val.E.) [14–17], has defined quality indicators for its breast cancer care network, which have been included in the 2018 P.Re.Val.E. Other indicators are under development and need to undergo further improvements before being permanently included within the P.Re.Val.E. indicators. These quality indicators were calculated with the data available at the regional level, based on scientific evidence provided by the national and international guidelines. The quality indicators reported in the current study assess the performance of breast units and LHUs within the surgery and post-surgery phases of the breast cancer clinical pathway.

A summary indicator for evaluating the breast cancer clinical network has never been measured at the regional level. The P.Re.Val.E. has developed treemaps to describe hospital and LHU performance. The treemap structure has an intuitive design and displays information from both general and specific analyses, providing healthcare facilities with an operational tool for the timely monitoring of their quality of care. Seven different clinical areas were taken into account within the treemaps: cardiology, neurology, pulmonology, general surgery, cancer surgery, pregnancy and childbirth, and orthopaedics. The P.Re.Val.E. divided its indicators according to the clinical areas of the treemaps [14]. Each indicator and each area were represented by two different methods at the same time: a point-score estimate and a colour scale. A score was assigned to each clinical area based on the ranking of the indicators involved. The colour scale corresponds to the score ranking, and was used to provide an immediate visual representation of the quality assessment. Using the same methodology, the overall score and colour were calculated for each hospital and LHU. The purpose of this study is to design treemaps to represent a summary of the breast cancer care network, using quality indicators, monitoring trends and updating data activity.

## Study goals and objectives

The primary outcome of this study is to provide a description of breast units and LHUs in terms of their levels of performance, creating a summary indicator.

The secondary outcome is to compare breast units and LHUs within the Lazio region. Furthermore, the hospitals and LHUs were compared with the regional average, and the temporal trend of the individual structures was analysed, in order to obtain an overview of the regional breast cancer care network.

## Methodology

### Data sources

The information needed for the definition and calculation of indicators came from the databases of Lazio’s Department of Health Information System, which anonymized patients, conferring them a unique identification code thorough the use of an algorithm.

Data for selecting the patient cohort were collected using the Italian hospital Schedule Of Discharge (SDO). This is an administrative information system containing socio-demographic and clinical inpatient data (diagnoses, procedures), as well as intra-hospital transfers for all hospital admissions and discharges of the Lazio region. Eligibility and exclusion criteria were defined using ICD-9-CM codes [18].

The anonymous identification code was used as a key for the deterministic record-linkage procedure, in order to integrate data taken from the SDO with additional data captured from the following administrative information systems:

- ISOSA (Information System of Outpatient Specialist Assistance): contains information on the regional service of outpatient specialist assistance, socio-demographic characteristics of patients (gender, age, place of residence) and clinical information, defined according to the national nomenclature of outpatient specialist assistance [19].
- ISHE (Information System of Health Emergency): is an integration of the SDO, including socio-demographic and clinical information about casualty patients, admissions and treatments.
- DIS (Drug Information System): contains information on prescriptions provided to Lazio residents, identified through the ATC (Anatomical Therapeutic Chemical) classification system [20].
- Tax register: includes information on life situation.

In this way, we recreated socio-demographic and health-related patient profiles, tracing the clinical history of the five years preceding the index admission.

### Ethics

This study was carried out in accordance with the current privacy policy on personal information in Italy and the results are reported as an aggregate. The Department of Epidemiology (DEP) is regulated by the Lazio Regional Health Service in managing and analysing data from the administrative information systems for epidemiological research, according to current regional law (Lazio, Italy). The DEP has access to anonymized data taken from the Regional Health Information Systems and it is not possible to trace patient identity. The DEP works in synergy with the Directorate for Health and Social Care Integration of the Lazio Regional Health Service. As a result, the DEP is entitled to use the data provided by the Health Information System Unit of the Lazio region for health and scientific purposes. This article reports on research developed within the P.Re.Val.E. [14–17]. Furthermore, the DEP has been identified as being responsible for the development of the P.Re.Val.E. and for all aspects related to its technical and scientific implementation, according to current regional law. For these reasons, it is exempt from the requirement for approval by an ethics committee in this setting.

### Study design and population

We created eight indicators suitable for assessing the quality of care. The scores for these indicators were calculated using healthcare data available at the regional level. The definition and selection of indicators were based on national standards and on evidence reported within international guidelines. P.Re.Val.E. has developed the following four indicators to evaluate the malignant breast cancer (MBC) surgery phase, with the first three as health outcome indicators: 1) volume of MBC surgery admissions; 2) proportion of surgeries performed in wards with a high volume of activity; 3) proportion of new resection surgeries; 4) proportion of reconstruction or implant-insertion surgery (Table 1). The volume of admissions for MBC surgery measures the number of MBC surgical procedures. Scientific evidence for the quality of breast cancer care shows an association between high volumes of activity and improved clinical outcomes [21–24]. According to Italian law [4] and the ESMO guidelines [6], every breast unit should admit at least 150 new cases every year. Hospital wards with an activity volume higher than 135 surgeries per year (threshold of 150 decreased by a tolerance of 10%) were identified as high activity volume. The indicator for new resection surgery measures the proportion of new surgeries performed within 120 days from breast-conserving surgery. this is based on evidence that reoperation after breast-conserving surgery results in worse cosmetic outcomes and causes additional stress for patients [5]. The proportion of reconstruction or implant-insertion surgeries in admissions for destructive surgery for invasive breast cancer is a process indicator. This is based on evidence that simultaneous breast destruction and reconstruction results in cosmetic satisfaction and better quality of life [5].

**Table 1:** The main characteristics of the indicators.

Code	Definition	Numerator	Denominator	Year of the index admission	Follow-up	Data sources
107	Volume of MBC surgery admissions	-	-	2017	-	SDO
556	Proportion of surgeries performed in wards with a volume of activity higher than 135 surgical interventions per year	Number of surgeries performed in wards with a volume of activity higher than 135 surgical interventions per year	Number of admissions for MBC surgery	2017	-	SDO
605	Proportion of new resection surgeries within 120 days of conserving surgery for MBC	Number of admissions with new resection surgeries within 120 days of conserving surgery for MBC	Number of admissions for resection surgery for MBC	2017	120 days from surgery	SDO, ISHE, ISOSA, tax register
606	Proportion of reconstruction or implant-insertion surgeries in admissions for demolitive surgery for MBC	Number of admissions with reconstruction or implant-insertion surgeries	Number of admissions for resection surgery for MBC	2017	the index hospitalization	SDO, ISHE, ISOSA, tax register
608	Proportion of patients who underwent mammography in the 18 months following discharge after MBC surgery	Number of admissions with a mammography undergone in the 18 months following discharge after MBC surgery	Number of admissions for resection surgery for MBC	2015	18 months from discharge	SDO, ISHE, ISOSA, tax register
609	Proportion of patients who received intensive follow-up in the 12 months following discharge after MBC surgery	Number of admissions with intensive follow-up in the 12 months following discharge after MBC surgery	Number of admissions for resection surgery for MBC	2016	12 months from discharge	SDO, ISHE, ISOSA, tax register
611	Proportion of patients starting medical therapy within 60 days of MBC surgery	Number of admissions with the first medical therapy within 60 days of MBC surgery	Number of admissions with surgical operation for MBC	2016	60 days from surgery	SDO, ISHE, ISOSA, tax register, DIS
613	Proportion of patients undergoing medical therapy who started radiotherapy within 365 days of conserving surgery for MBC	Number of admissions with the first radiotherapy within 365 days and the first medical therapy within 180 days of conserving surgery for MBC	Number of admissions with the first medical therapy within 180 days of conserving surgery for MBC	2015	365 days from surgery	SDO, ISHE, ISOSA, tax register, DIS

MCB, malignant breast cancer.

P.Re.Val.E. has developed the following four process indicators to evaluate the post-surgery phase (Table 1): 1) The proportion of patients who underwent mammography in the 18 months following discharge after MBC surgery measures the proportion of new cases receiving an active follow-up, aimed at early identification of disease recurrence [7,8]. 2) The proportion of patients who received intensive follow-up in the 12 months following discharge after MBC surgery, aimed at detecting redundant combinations of diagnostic procedures used to identify disease recurrence [5]. 3) The proportion of patients starting medical therapy within 60 days of MBC surgery measures the timeliness in starting medical therapy. The timeliness of medical intervention is associated with an increased probability of survival in the medium to long term [5,7,8]. 4) The proportion of patients undergoing medical therapy who started radiotherapy within 365 days of conserving surgery for MBC measures the use of post-operative complementary radiotherapy, which decreases the local recurrence risk and increases the probability of long-term survival [5,7,8].

Table 1 reports the descriptions of the quality indicators and the respective health information systems used for calculating scores. The cohort study population for the volume indicators was characterized by inpatients and day-hospital patients, who were residents of the Lazio region, admitted to regional hospitals with a primary or secondary diagnosis of MBC (malignant neoplasm of female breast and carcinoma in situ of breast ICD-9-CM [18] 174, 233.0) and a primary or secondary intervention of demolitive surgery (ICD-9-CM 85.33, 85.34, 85.35, 85.36, 85.4.x) or conservative surgery (ICD-9-CM 85.2x) in the recruiting period. The populations for the other indicators were specific sub-cohorts of the volume indicators’ population, in accordance with the eligibility and exclusion criteria (reported in S1 and S2 Tables), which differ for each indicator. Details of the make-up of the indicators are reported in the supporting information (S3–S6 Tables). The analyses were performed on an annual basis, by the LHU of residence (regardless of the structure of discharge) and by the hospital which performed the surgical intervention. The annual regional average of each indicator was also calculated. The year of analysis changes along with the indicator, according to data availability from the different health information systems. The first four indicators of Table 1 were updated for the year 2017, while the other indicators, given the long follow-up periods or the older data sources, are limited to 2015 or 2016.

### Treemap design

Treemaps were used to visualize a summary of the breast cancer care network within hospitals and LHUs. Two main areas were identified: surgery and post-surgery. The indicators reported in Table 2 were selected to represent these areas.

**Table 2:** The areas and thresholds of the treemap indicators.

Area	Indicator	Level of performance				
		Very High	High	Average	Low	Very low
		1	2	3	4	5
		deep green	green	yellow	orange	red
Surgery	Proportion of surgeries performed in wards with a volume of activity higher than 135 surgical interventions per year	= 100	100 l– 80	80 l–50	50 l–30	< 30
Surgery	Proportion of new resection surgeries within 120 days of conserving surgery for MBC	< 5	5 –l 8	8 –l 12	12–18	> 18
Surgery	Proportion of reconstruction or implant-insertion surgeries in admissions for demolitive surgery for MBC	> 60	-	60–40	-	< 40
Post-surgery	Proportion of patients who underwent mammography in the 18 months following discharge after MBC surgery	>70	70–64	64 l– 57	57 l– 50	< 50
Post-surgery	Proportion of patients who received intensive follow-up in the 12 months following discharge after MBC surgery	< 10	10 –l 20	20 –l 30	30–50	> 50
Post-surgery	Proportion of patients starting medical therapy within 60 days of MBC surgery	> 70	70–60	60 l– 40	40 l– 30	< 30
Post-surgery	Proportion of patients undergoing medical therapy who started radiotherapy within 365 days of conserving surgery for MBC	> 87	87–79	79 l– 70	70 l– 50	< 50

MCB, malignant breast cancer.

In order to allow aggregation of scores for different indicators belonging to the same clinical area, up to five evaluation classes were defined, by assigning all indicators with a score ranking from 1 (very high performance, in dark green) to 5 (very low performance, in red). The P.Re.Val.E. had already identified classes for ward volume and new resection surgery indicators [14]. Threshold values for the indicator classes (shown in Table 2) were defined using the "Jenks natural breaks" algorithm [25]. Each indicator was visualized in a block which reports the score and the proportional colour assigned. The blocks were also used to represent the surgery and post-surgery areas: arrows indicated the colour scale for these areas, using the same colours used to define the individual indicators into classes. The mean score ranking of the indicators gave the summary score and colour to each area. The volume of activity was used as an *a priori* evaluation criterion: hospitals with a volume below 135 surgical interventions per year obtained the lowest rating class for the surgery area, regardless of their indicator values. The mean rankings of the clinical areas gave pooled results for each structure and LHU, in order to obtain a summary description of the clinical assistance provided. The treemap score rankings were used to describe and monitor differences in performance.

### Definition and attribution of outcomes

Outcomes were measured by the summary indicators and quality indicators. The results are attributable to the admitting structure, which performs the surgical interventions, or to the LHU of residence. The outcome is the breast unit and LHU performance, in terms of volume of activity, performance of surgical interventions, post-surgery assistance and timeliness of medical therapy or radiotherapy beginning.

### Sample size

For each indicator, the DEP calculated a minimum size per structure, which allowed expected effects to be evaluated as statistically significant, expressed as the ratio between the observed risk in the single structure (p_i_) and the observed risk in the overall Lazio region population (π).

Minimum size per structure = (Z_α_ + Z_β_)^2^ / [2 × arcsin (√π) - 2 × arcsin (√p_i_)]^2^.

In this formula, Z_α_ is the value from the standard normal distribution at α (type I error), Z_β_ is the value from the standard normal distribution at β (type II error).

The hypotheses on the expected effects, calculated in terms of risk ratio (RR), were differentiated according to the frequency of the study outcomes in the overall population:

- a RR of 4 was considered for outcomes with a frequency of less than 1%;
- a RR of 3 was considered for outcomes with a frequency between 1% and 5%;
- a RR of 2 was considered for outcomes with a frequency of higher than 5%.

Based on the described formula, a minimum size per structure for each indicator was calculated, obtaining 25 as the lowest value. For this reason, we calculated the indicator only if the denominator of the single structure reached a minimum number of 25 admissions for MBC surgery. This allows to evaluate as statistically significant all the proportions of the indicators which reached the minimum size per structure. The power (1-β) of the test is set at 80%, the significance (α) at 5%.

### Data management and statistical analysis

For each indicator reported in Table 2, calculation of the regional average, as well as analysis by hospital of discharge and by LHU of residence, was performed. The integration of different information systems was performed using the record linkage technique. The construction of the predictive model was assessed through multivariable logistic regression. The potential predictors, identified based on the available literature, were selected by a stepwise procedure. The threshold of p-value = 0.05 was used for variable selection and elimination. The risk ratio was used to express the comparison between the analysis by structure and by LHU with the regional average. The risks were adjusted using the previously defined predictive model; the main covariates included in the multivariate models were: age, histological type, adjuvant therapy and typology of admission. For each indicator reported in Table 2, the adjusted risk ratio estimated for each hospital/LHU was achieved by direct standardization, in relation to the total Lazio population registered in the corresponding year of the analysis. The direct standardization method enables calculation of the expected risks in the event that all the structures and all the LHUs present the same distribution of the Lazio region population. The expected risks thus obtained were corrected through a multiplicative factor, which takes into account the non-linear nature of the model used. For each indicator, the temporal trend was developed by each hospital and LHU. The time trend analysis was carried out by including, in a single adjustment model, the interaction term between hospital/LHU and year of analysis. This ensures complete comparability among the estimates obtained for different years. Therefore, the time trend was adjusted for all the potential confounding factors identified by the predictive model. Statistical analyses were performed using SAS ® (version 9.4) statistical software. The thresholds of the evaluation classes for the indicators, reported in the treemaps, were defined using the "Jenks natural breaks" algorithm [25]. This is a classification method which determines the best arrangement of values into different clusters, based on natural groupings inherent in the data. The natural breaks identified thresholds for dividing values in order to minimize differences within the same classes and maximize differences between classes. The thresholds were set in accordance with relatively large differences in the values. The D3.js JavaScript graphic library was used for the visualization of the treemaps.

## Results

In 2017, 6218 surgical interventions for MBC were performed in the hospitals of the Lazio region, and the proportion of interventions carried out in breast units was 71.2%. Only 15 hospitals in 2017 reached the activity volume of 135 interventions per year (S7 Table) and most of them are located in the metropolitan area of Rome. Three hospitals, although not formally identified as breast units, over the last few years achieved high volume of activity. Regarding the number of resident hospitalizations within the structures of the Lazio region, in 2017, 76.4% occurred in two of Rome’s LHUs (41.4% in the RM1 LHU and 35% in the RM2 LHU). The remaining admissions took place in the other 8 LHUs of the region.

The proportion of surgeries performed in wards with a volume of activity higher than 135 surgical interventions per year progressively increased from 2012 (39.5%), reaching 58.6% in 2017. Despite this, data for 2017 showed a considerable intra-hospital fragmentation: some breast units carried out a high proportion of surgical interventions for MBC in wards with a low volume of activity, which did not reach the expected threshold. One example was the S. Filippo Neri hospital, which, in 2017, achieved a volume of activity of 176 surgeries, but none of the interventions was performed in wards with a high volume of activity.

Starting from 2010, a statistically significant increase of 2.72% per year (p-value = 0.0001) was observed in the proportion of reconstruction or implant-insertion surgeries in admissions for demolitive surgery for invasive breast cancer, reaching 56.6% in 2017. Despite this, there was heterogeneity among hospitals, ranging between 38.9% (adjusted RR = 0.69; p-value = 0.02) and 67.6% (adjusted RR = 1.19; p-value = 0.005).

The proportion of new resection surgeries within 120 days of conserving surgery for MBC progressively decreased in the years from 2010 (13.7%) to 2016 (6.8%). In 2017, the proportion remained stable compared with the previous year, reaching a value of 7.4%. One of the highest proportions was observed in the S. Filippo Neri hospital (19.3%; adjusted RR = 2.61; p-value = 0.0001). This hospital showed an increase compared with the previous year, from 11.6% in 2016 to 26.4% in 2017 (adjusted RR = 2.27; p-value: 0.022).

The proportion of patients who underwent mammography in the 18 months following discharge after MBC surgery showed a progressive decline, starting from 2011 (56.7%) and reaching 52.8% in 2015. Data for 2015 showed variability for LHUs of residence: the highest value was 73.7% (adjusted RR = 1.4; p-value = 0.0001) and the lowest value was 46% (adjusted RR = 0.87; p-value = 0.017).

The proportion of patients who received intensive follow-up in the 12 months following discharge after MBC surgery showed a slight reduction over the years, from 22.1% in 2009 to 18.2% in 2016. The data for 2016 showed heterogeneity in LHUs of residence, the highest value being 24.3% (adjusted RR = 1.33; p-value = 0.001) and the lowest 8.6% (adjusted RR = 0.47; p-value = 0.0001).

From 2010, there was a statistically significant decrease of 1.04% per year (p-value = 0.0001) in the proportion of patients starting medical therapy within 60 days of MBC surgery, reaching a value of 57.1% in 2016. The data for 2016 showed variability in LHUs of residence, with a range between 68.7% (adjusted RR = 1.20; p-value = 0.004) and 35.2% (adjusted RR = 0.62; p-value = 0.0001).

The proportion of patients undergoing medical therapy who started radiotherapy within 365 days of conserving surgery for MBC remained stable in the years 2010 (73.9%) to 2014. In 2015, a slight increase was observed, reaching 77.1%. The data for 2015 showed heterogeneity in LHUs of residence, with the highest value for the Viterbo LHU (90.4%; adjusted RR = 1.17; p-value = 0.0001) and the lowest value for the RM6 LHU (57%; adjusted RR = 0.74; p-value = 0.0001).

Performance was assessed taking the selected indicators and the care network into account. The overall score was expressed on the basis of the established classes. The reconstruction indicator was tested by including the crude score for hospitals with missing values. In all the analyses mentioned, the pooled rankings never changed.

**Fig 1:**
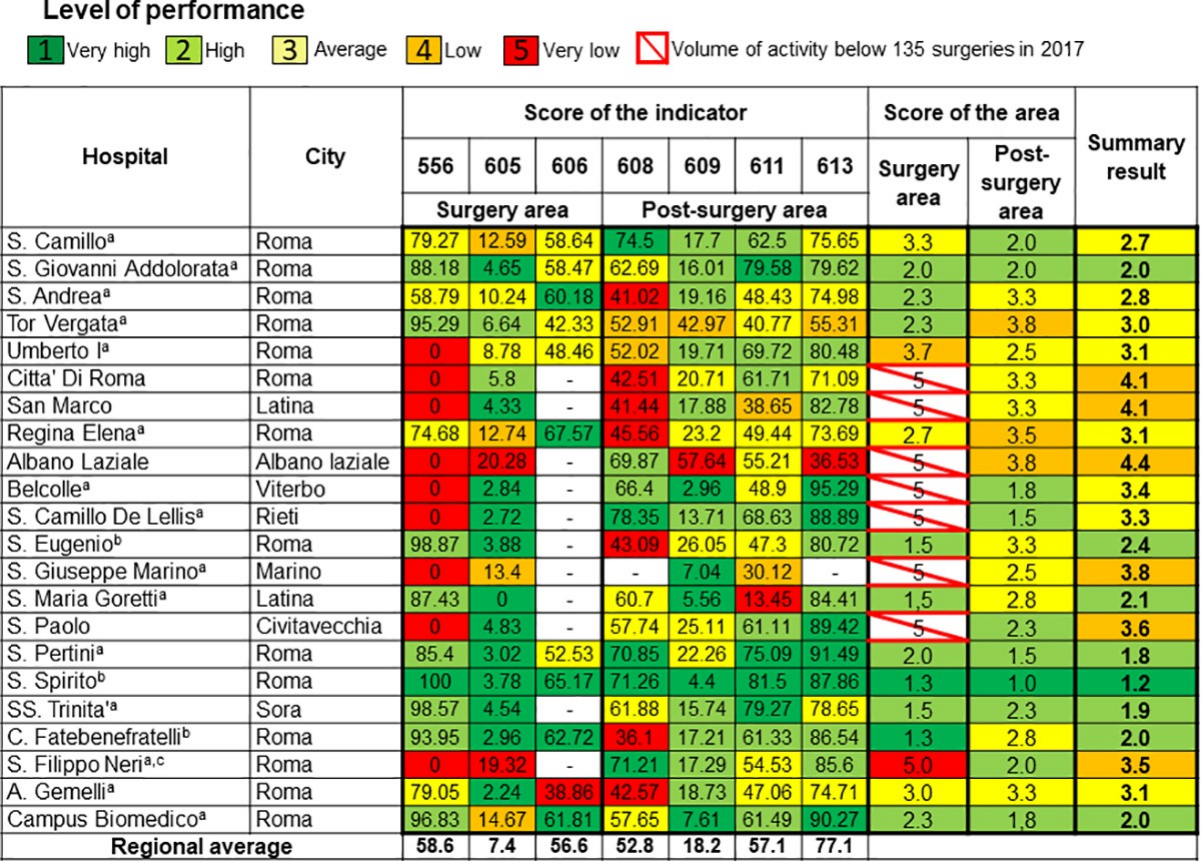
Treemap of the results for hospitals. ^a^ breast unit. ^b^ hospitals with the appropriate requirements and a high volume of activity. ^c^ the verification of the input data is required. 556, Proportion of surgeries performed in wards with a volume of activity higher than 135 surgical interventions per year. 605, Proportion of new resection surgeries within 120 days of conserving surgery for malignant breast cancer. 606, Proportion of reconstruction or implant-insertion surgeries in admissions for demolitive surgery for malignant breast cancer. 608, Proportion of patients who underwent mammography in the 18 months following discharge after malignant breast cancer surgery. 609, Proportion of patients who received intensive follow-up in the 12 months following discharge after malignant breast cancer surgery. 611, Proportion of patients starting medical therapy within 60 days of MBC surgery. 613, Proportion of patients undergoing medical therapy who started radiotherapy within 365 days of conserving surgery for MBC.

**Fig 2:**
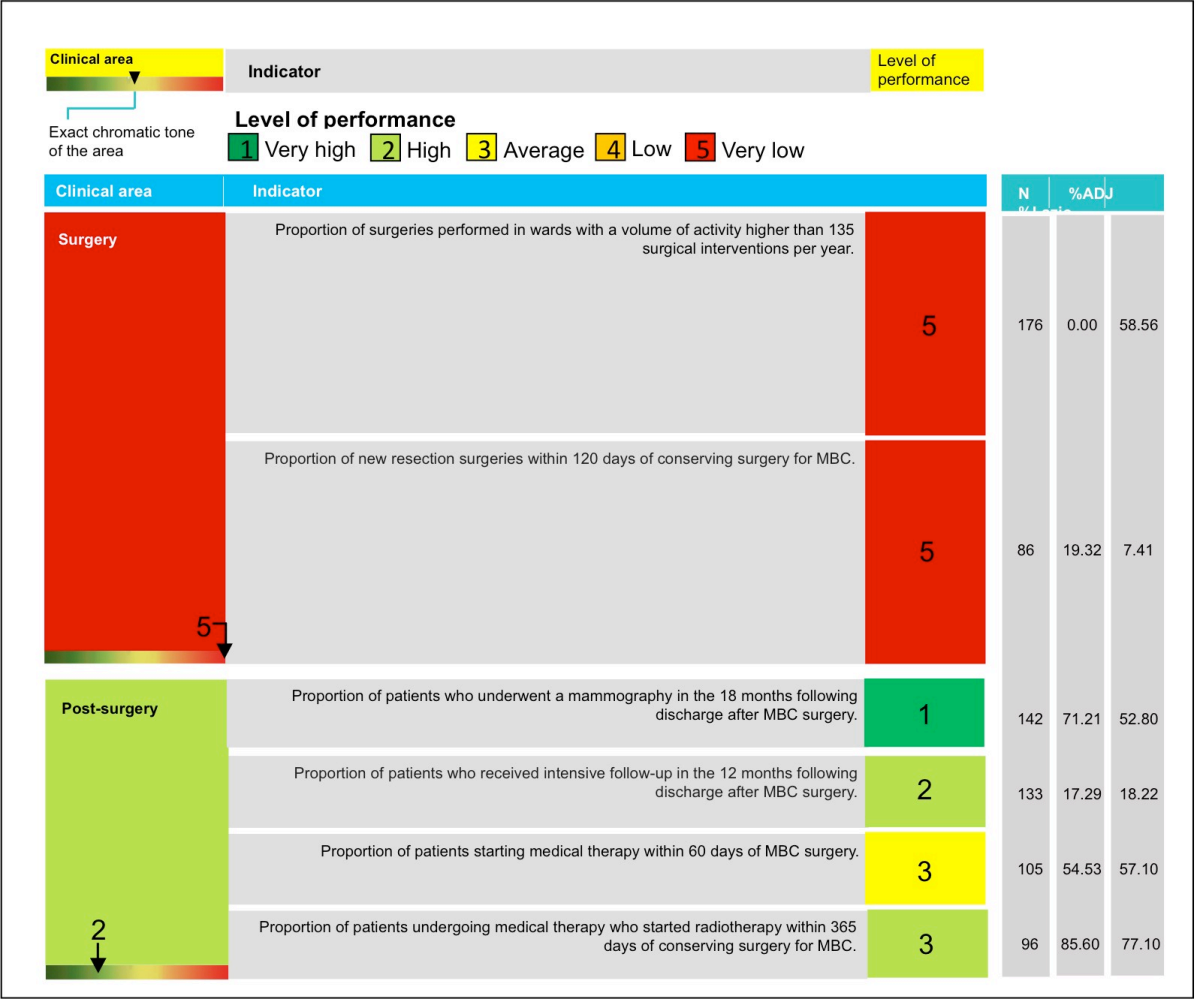
Treemap details for the San Filippo Neri hospital. MBC, Malignant Breast Cancer.

The final results showed heterogeneity among hospitals (Fig 1), with the best score (very high performance) in both clinical areas for the S. Spirito hospital, which reached the highest level of performance. Seven hospitals did not reach the threshold of 135 surgical interventions per year (S7 Table). As a result, they achieved the lowest rating class for the surgery area, regardless of their indicator values. Considering all of the 22 hospitals, eight achieved a high level of overall performance. The S. Filippo Neri (Fig 2) and S. G. Marino breast units did not reach an average level of overall performance and their results were significantly different from the regional average.

The DEP audited the S. Filippo Neri hospital in order to assess the quality of the data provided on surgeries. Verification of the data input for the indicator on the volume of wards, resulted in the incorrect attribution of surgical interventions to different wards, although in most cases the surgery team was the same. After the data was corrected, the proportion of the indicator was higher than 90%. For this reason, it was re-evaluated according to the score ranking, and the S. Filippo Neri hospital obtained a high level of performance for this indicator. The audit of data quality for the new resection surgery indicator revealed correct coding of the input data. For this reason, the score for the new resection surgery indicator did not change. As a result, the score for the surgery area shifted to the next class up (level of performance from very low to low, reaching a score of 3.5) and the summary ranking of the hospital resulted in an average level of performance (summary score = 2.8).

**Fig 3:**
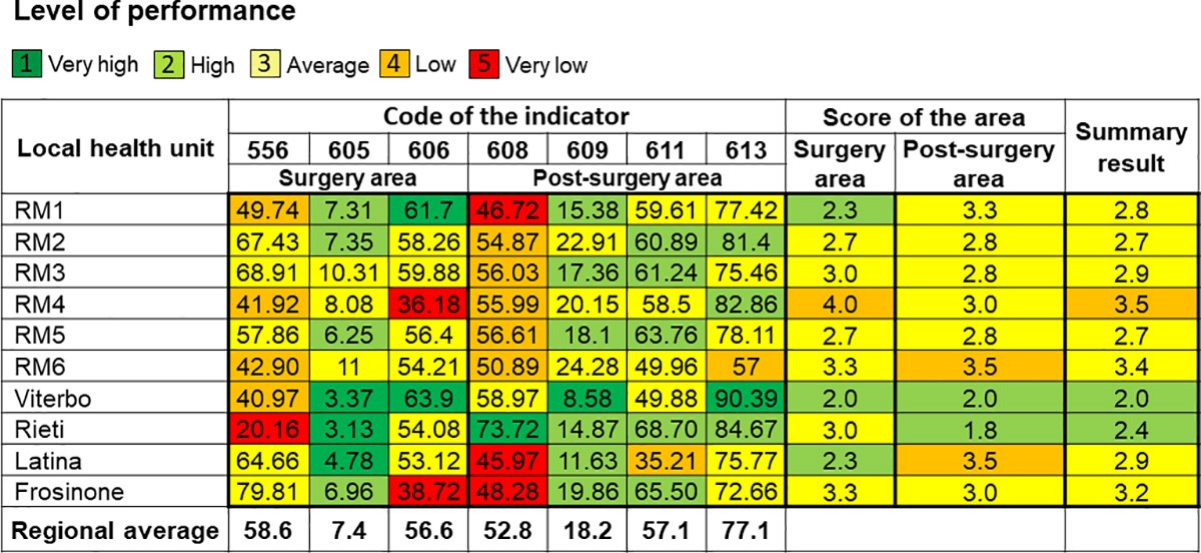
Treemap of the results for LHUs. 556, Proportion of surgeries performed in wards with a volume of activity higher than 135 surgical interventions per year. 605, Proportion of new resection surgeries within 120 days of conserving surgery for malignant breast cancer. 606, Proportion of reconstruction or implant-insertion surgeries in admissions for demolitive surgery for malignant breast cancer. 608, Proportion of patients who underwent mammography in the 18 months following discharge after malignant breast cancer surgery. 609, Proportion of patients who received intensive follow-up in the 12 months following discharge after malignant breast cancer surgery. 611, Proportion of patients starting medical therapy within 60 days of MBC surgery. 613, Proportion of patients undergoing medical therapy who started radiotherapy within 365 days of conserving surgery for MBC.

The LHUs showed less variability in level of performance than the hospitals. All of the LHUs achieved at least an average overall performance, except for RM4 LHU. The Latina and Rieti LHUs reached a high level of overall performance (Fig 3).

## Discussion

The current study describes the breast cancer care network within the Lazio region, providing an overview of the performance level of hospitals and LHUs. This is the first attempt to apply the treemap logic to a single medical specialty, in order to obtain a summary indicator for the evaluation of a care network at the regional level. The treemap constitutes an interactive tool, which can be displayed through the P.Re.Val.E. website [14]. Each treemap has an intuitive graphic design and its online version is provided with many details (Fig 2), which supply information about the indicators’ results, including cohort size and the reference to the regional average.

In 2017, seven hospitals did not reach the volume of 135 MBC surgeries per year (S7 Table) and the proportion of surgeries performed in wards with a volume of activity higher than 135 surgical interventions per year reached 58.6%, showing an intra-hospital fragmentation. This value is indeed lower than both the national average (69.6%) [26] and the results documented by Rubio et al. [24] (70%). Amato et al. [21] have reported that in Italy hospitals and wards with high volume of activity offer better clinical outcomes. The same association has been reported in other international studies [22,23]. In accordance with Rubio et al [24] we consider too low the minimum EUSOMA standard of 50 new cases/year. National law [4], the UEMS eligibility criteria for European Board Examinations for the qualification as Fellow of European Board of Surgery in Breast References Surgery [27] and the ESMO guidelines [6] consider a minimum of 150 new MBC surgeries per year. We used this threshold as *a priori* evaluation criterion, decreasing it by a tolerance of 10% and considering all the performed surgeries, not only the new cases. This criterion ensures the attribution of high level of performance to the excellence centres.

The proportion of immediate reconstruction progressively increased from 2010, consistently with the study reporting on 2315 patients of a single European institution of Sardinia (another Italian region) [28]. This proportion reached 56.6% in 2017, largely overcoming the minimum EUSOMA standard of 40% [6] and the national average [26]. The proportion of new resection surgeries within 120 days of conserving surgery for MBC decreased in the last years, reaching 7.4% in 2017. This proportion is lower than the national average [26] and EUSOMA minimum standard [5]. The proportion of patients who underwent mammography in the 18 months following discharge after MBC surgery reached 52.8% in 2015: a lower proportion with respect to the AIOM indicator [8]. This result is just limited to identifying mammography provided by the national health system. The proportion of patients who received intensive follow-up in the 12 months following discharge after MBC surgery reached 18.2% in 2016, it is a high proportion considering that EUSOMA reported the absence of survival benefit from intensive screening for secondary prevention in patients with asymptomatic metastatic diseases. The medical therapy and radiotherapy indicators, in 2016 and 2015 respectively, reached low values compared with the EUSOMA standards [5] and most of the international studies which have assessed the use of adjuvant therapy after MBC surgeries [29–34].

A health care system should be sustainable and equitable and should be based on the available scientific evidences [5]. The adherence to guideline in breast cancer clinical practice is a fundamental requirement to ensure high quality of care [8]. We assessed the care quality within the regional context, comparing breast units and LHUs within Lazio.

The thresholds of the evaluation classes for the treemap indicators (Table 2) was set using the "Jenks natural breaks" [25]; this method has been already used by Di Martino et al. [35] for creating evaluation classes. This algorithm is based on data value distribution and allow data-specific classification and it is useful for comparing data which came from the same local context. The thresholds are dynamic elements, the classification needs to be periodically updated, in order to assure the best arrangement of values. This method provides healthcare professionals with a challenging tool, promoting the progressive improvement in the quality of care.

The pooled score ranking showed high variability in the level of performance for hospitals, with a consequent inequality in the care provided (Fig 1). Three of the seven structures not identified as breast units (according to the DCA 38/2015 [8]), reached a high level of overall performance and all the requirements for carrying out this activity. This was especially the case of the S. Spirito hospital, which obtained the highest score ranking in all the indicators, showing his capability to provide high quality of care within the regional framework. Most of the breast units obtained an average level of performance and one third of them stood out for their high overall performance. The S. Filippo Neri breast unit achieved low overall performance and for this reason, it was subjected to further analysis. The S. G. Marino breast unit resulted in a summary low level of performance. Nevertheless, this hospital showed a progressive improvement in the last years.

The treemaps allowed the evaluation of LHU’s performance. The LHUs have the task of managing the network, which includes the coordination of the overall services provided to the resident of the geographic area under their authority [3]. Most of the LHUs of Lazio guaranteed a valid network of care within their territory (Fig 3). The Viterbo and Rieti LHU proved to have a better management than the other provincial LHUs. The situation of Rome’s LHUs was different due to a lack of territorial boundaries: some LHUs had the capability not only to answer the needs of their residents, but also of other LHUs’ residents, which do not have a specific breast unit within their territory.

We assessed providers’ performance within the breast cancer care network, considering hospitals and LHUs as key facilities. The breast cancer network consists of a very complex framework: beside breast units and LHUs, breast cancer treatment involves screening centres, clinical diagnostic facilities and general practitioners [9]. Furthermore, the breast cancer clinical pathway includes many clinical phases, from screening for primary prevention, to palliative care. The DEP does not have access to the clinical data necessary for the evaluation of the entire pathway and all the facilities involved in the breast cancer network. Information about the stage of cancer or patients-reported data are not systematically collected at the regional level and are not included in the SDO or in other Lazio’s administrative information systems. For these reasons the summary indicators and the quality indicators were calculated using administrative healthcare data available at the regional level. The treemaps could be reproduced and adapted for other local contexts using systematically collected administrative data, in order to limit inappropriateness and ensure uniform levels of breast cancer care within local areas.

Despite the important advantages in the use of administrative data for the performance assessment [17,36], a critical aspect is the quality of the data provided. Since the process of erroneous input data causes mistaken results [37], the verification of the data quality recorded in the health information systems is essential to ensure the correct management of the input data [38] involved in the calculation of the treemap indicators. The P.Re.Val.E. conducts audits of data quality in hospitals with specific results, which are significantly different from the regional average. The aim of the audits is to identify any critical issues, which may be subjected to specific improvement interventions.

An example of the audit concerns the S. Filippo Neri hospital, which reached a low level of performance for both the indicators of the surgery area (Fig 2). In the case of the indicator for the volume of the ward, the problem was the incorrect data encoding, and the hospital was urged to modify data entry modalities. For this reason, the indicator was re-calculated and re-evaluated, with a subsequent improvement of the level of performance. The indicator for new resection surgeries showed a different scenario, in which the audit found a correct coding of the input data. In this case, the P.Re.Val.E. recommended the hospital to carry out an internal organizational audit. The reason for low performance was a clinical choice: the surgeon considered the clinical examination of the patients after 30 days of the intervention and, in case of necessity, the consequent new-resection surgery. This pattern of care constitutes a deviation from the quality standards established by the guidelines.

The effectiveness of public disclosure of performance data or on regulatory interventions is subject to debate [16, 39–42]. The next step is the evaluation of audit and feedback interventions to improve the quality of care and to guarantee homogeneous levels of care throughout the region.

## Conclusions

This study is a pilot implementation of a summary indicator, displayed through the treemap and determined by the pooled results from the surgery and post-surgery areas, for the evaluation of the performance within the breast cancer care network. The treemaps provide data with different levels of detail and so it is a suitable instrument for heterogeneous users. The treemaps provide cues to fully understand the differences and to gather useful information. In fact, the analysis of the best clinical network and critical issues offers a valuable opportunity for decision makers’ reflection, in order to adopt the best strategy of action for the implementation of the breast cancer care network. By processing the treemaps, the P.Re.Val.E. supplies the politician, clinicians and managers of the Lazio region with a transparent instrument of governance, in order to direct efforts for the improvement and to promote homogeneity in the quality of care.

## Supporting information

S1 Table Eligibility criteria. (DOCX)

S2 Table Exclusion criteria. (DOCX)

S3 Table Outcomes of interest. (DOCX)

S4 Table Potential confounders. (DOCX)

S5 Table Severity factors. (DOCX)

S6 Table Predictive model. (DOCX)

S7 Table Activity volume of the hospitals. (DOCX)
