# Supplementary material for: Evaluation of the breast cancer care network within the Lazio Region (Central Italy)
Source: PLoS One. 2020 Sep 3;15(9):e0238562. doi: 10.1371/journal.pone.0238562 (PMC7470269; doi:10.1371/journal.pone.0238562)
Supplement: S1 Table — (DOCX) [file pone.0238562.s001.docx]

**S1 Table. Eligibility criteria.**

| **ELIGIBILITY CRITERION** | **INDICATOR** | | | | | | |
| --- | --- | --- | --- | --- | --- | --- | --- |
|  | **556** | **605** | **606** | **608** | **609** | **611** | **613** |
| Admission | Ordinary/Day hospital | | | | | | |
| Region of residence | Lazio | | | | | | |
| Discharge date | 01/01/2010  31/12/2017 | 01/01/2010  31/08/2017 | 01/01/2010  30/11/2017 | 01/01/2010  31/12/2015 | 01/01/2010  31/12/2016 | 01/01/2010  31/12/2016 | 01/01/2010  31/12/2015 |
| ICD-9-CM Diagnosis codes | 174, 198.81,  233.0 | 174, 233.0 | 174 | 174, 233.0 | | 174 | 174, V58.1 |
| ICD-9-CM Procedure codes | 85.2x, 85.33, 85.34, 85.35, 85.36, 85.4.x | 85.2x | 85.33, 85.34, 85.35, 85.36, 85.4.x | 85.2x, 85.33, 85.34, 85.35, 85.36, 85.4.x | | | 85.2x, 99.25, 99.28 |
| ATC classification system codes | - | - | - | - | - | - | L01,  L02 |
| National nomenclature  codes | - | - | - | - | - | - | 99.24.1, 99.25 |
