# Supplementary material for: Evaluation of the breast cancer care network within the Lazio Region (Central Italy)
Source: PLoS One. 2020 Sep 3;15(9):e0238562. doi: 10.1371/journal.pone.0238562 (PMC7470269; doi:10.1371/journal.pone.0238562)
Supplement: S2 Table — (DOCX) [file pone.0238562.s002.docx]

**S2 Table. Exclusion criteria.**

| **EXCLUSION**  **CRITERION** | **INDICATOR** | | | | | |
| --- | --- | --- | --- | --- | --- | --- |
|  | **605** | **606** | **608** | **609** | **611** | **613** |
| Gender | Male | | | | | |
| Age | <18 or >100 years | | | | | |
| ICD-9-CM Diagnosis codes in the index admission | 198.81 | | | | | - |
| ICD-9-CM Procedure codes in the index admission | 85.3x,  85.4.x | - | - | - | - | - |
| ICD-9-CM Diagnosis codes in the index admission and in the previous 5 years | V42.0, V42.1, V42.2, V42.6, V42.7 | | | | | |
| ICD-9-CM Procedure codes  in the index admission and in the previous 5 years | 01.2, 01.3, 01.4, 01.5, 02, 33.5,  33.6, 35, 36, 37.5, 43.9, 45.8, 50.4, 50.5, 52.7, 52.8, 55.5, 55.6, 57.7 | | | | | |
| ICD-9-CM Diagnosis codes in the previous 5 years and up to 6 months before the surgical intervention | 174, 198.81, 233.0 | | | | | |
| ICD-9-CM Procedure codes  in the previous 5 years before the admission | 85.2x, 85.3x, 85.4.x | | | | | |
| Codes  in the previous 6 months before the admission | - | - | - | - | ICD-9-CM diagnosis codes: V58.1.  ICD-9-CM procedure codes: 99.25, 99.28.  ATC classification system codes: L01, L02.  National nomenclature codes: 99.24.1, 99.25 | - |
| ICD-9-CM Procedure codes within 4 months of surgery | - | - | - | - | 85.20-85.25, 85.33-85.36, 85.41-85.48 | - |
| Treatment within 6 months of surgery | - | - | - | - | Radiotherapy without medical therapy | - |
| ICD-9-CM Diagnosis codes within 12 months of discharge | - | - | - | 197, 198, 199 | - | - |
| ICD-9-CM Diagnosis codes in the index admission and within 12 months of discharge |  |  |  |  |  | 197, 198, 199 |
| Died within the follow-up period | Within 120 days of surgery | During the index  hospitalization | Within 18 months of discharge and without mammography | Within 12 months of discharge | Within 60 days of surgery and without medical therapy | Within 365 days of surgery and without radiotherapy |
