# Supplementary material for: Evaluation of the breast cancer care network within the Lazio Region (Central Italy)
Source: PLoS One. 2020 Sep 3;15(9):e0238562. doi: 10.1371/journal.pone.0238562 (PMC7470269; doi:10.1371/journal.pone.0238562)
Supplement: S3 Table — (DOCX) [file pone.0238562.s003.docx]

**S3 Table. Outcomes of interest.**

| **OUTCOME OF INTEREST** | **INDICATOR** | | | | | | | |
| --- | --- | --- | --- | --- | --- | --- | --- | --- |
|  | **556** | **605** | **606** | **608** | **609** | **611** | **613** |  |
| Description | - | - | - | - | Combination of the following diagnostic procedures: bone scan and cancer markers and (computed axial tomography or magnetic resonance imaging of abdomen/  thorax or  abdominal ultrasound) | - | - |  |
| ICD-9-CM Diagnosis codes | - | - | - | - | - | V58.1 | V58.0 |  |
| ICD-9-CM Procedure codes | - | 85.2x, 85.3x,  85.4.x | 85.33,  85.35,  85.53,  85.54  85.7,  85.85,  85.95 | 87.37 | 92.14, 92.18, 88.01, 88.02, 87.41, 88.95, 88.74-88.76 | 99.25, 99.28 | 92.2x |  |
| National nomenclature codes | - | - | - | 87.37.1, 87.37.2 | 92.18.2, 92.18.7, 99.55.x (except 99.55.4), 90.56.3, 88.01.1-88.01.5, 88.95.1-88.95.7 (except 88.95.3, 88.95.6), 87.41, 87.41.1, 88.74.1-88.74.6, 88.75.1, 88.75.2, 88.76.1, 88.78.1, 88.78.2, 88.79.7 | 99.24.1,  9.25 | 92.2x, 92.47.8,  92.47.9 |  |
| ATC classification system codes | - | - | - | - | - | L01, L02 | - |  |
| Ward | Volume of activity more than 135 surgeries per year | - | - | - | - | - | - |  |
