# Supplementary material for: Evaluation of the breast cancer care network within the Lazio Region (Central Italy)
Source: PLoS One. 2020 Sep 3;15(9):e0238562. doi: 10.1371/journal.pone.0238562 (PMC7470269; doi:10.1371/journal.pone.0238562)
Supplement: S4 Table — (DOCX) [file pone.0238562.s004.docx]

**S4 Table. Potential confounders.**

The age measured in years was identified as potential confounder for all the indicators. The following comorbidities were considered for the 605 and 606 indicators.

| **POTENTIAL CONFOUNDERS** | **ICD-9-CM codes** | |
| --- | --- | --- |
| **Description** | **In the index admission** | **In the previously 5 years or in the emergency room admission** |
| Diabetes | - | 250.0-250.9 |
| Obesity | 278.0 | 278.0 |
| Lipid metabolism disorder | 272 | 272 |
| Anaemia | 280-284, 285 (except 285.1) | 280-284, 285 (except 285.1) |
| Clotting disorder | 286 | 286 |
| Others blood diseases | 287-289 | 287-289 |
| Arterial hypertension | - | 401-405 |
| Ischaemic heart disease | 412, 414 | 410, 412, 429.7, 411, 413, 414 |
| Previous coronary revascularisation | V45.81, V45.82 | 00.66, 36.1, 36.0, V45.81, V45.82 |
| Heart failure | - | 428 |
| Other heart condition /surgical intervention | 393-398, 423 (except 423.0), 424, 425, 745, 746.3-746.6, V15.1, V42.2, V43.2, V43.3 | 093.2, 391, 393-398, 420-425, 429 (except 429.7),  745, 746.3-746.6, V15.1, V42.2, V43.2, V43.3  procedures: 35, 37.0, 37.1, 37.3, 37.4, 37.5, 37.6, 37.9 |
| Arrhythmias | V45.0, V53.3 | 426.0, 426.10, 426.12, 426.13, 426.7, 426.9, 427, 785.0, 996.01, 996.04, V45.0, V53.3 |
| Cerebrovascular disease | 438  440-447 (except 441.0, 441.1, 441.3, 441.5, 441.6, 444, 445), 557.1, 093.0 | 430-438  procedures: 38.11, 38.12, 38.31, 38.32, 38.41, 38.42, 38.61, 38.62  440-447, 557, 093.0  procedures: 38.18, 38.38, 38.48, 38.68, 39.29  procedures: 38.14-38.16, 38.34-38.37, 38.44-38.47, 38.64-38.67, 39.52, 39.54 |
| Chronic obstructive pulmonary disease and allied conditions | - | 490-492, 493, 494, 496 |
| Chronic kidney disease | 582-583, 585-588, V42.0, V45.1, V56  Procedures: 38.95, 39.95, 54.98 | 582-588, V42.0, V45.1, V56  Procedures: 38.95, 39.95, 54.98, 55.6 |
| Moderate/severe liver disease | 456.0- 456.2, 571-572 (except 571.1, 572.0- 572.2), 573.0, V42.7 | 456.0- 456.2, 571-572, 573.0, V42.7 |
| Chronic inflammatory bowel disease | 555, 556 | 555, 556 |
| Pancreatic disease | 577.1-577.9 | 577.0-577.9 |
| Hemiplegia and others paralyses | 342, 344 | 342, 344 |
| M[ental and physical](https://context.reverso.net/traduzione/inglese-italiano/mental+and+physical) impairment | 290.0-290.4, 294.1, 331.0, 332, 261,262, 276.4, 276.5X, 799.4 | 290.0-290.4, 294.1, 331.0, 332 |
| Diseases of pulmonary circulation | - | 415-417 |
| Respiratory failure | - | 518.81, 518.82, 518.83, 518.84 |
| Others diseases of respiratory system | - | 515, 517 |
| Human immunodeficiency virus [HIV] disease/Asymptomatic HIV infection status | - | 042, V08 |
