# Supplementary material for: Evaluation of the breast cancer care network within the Lazio Region (Central Italy)
Source: PLoS One. 2020 Sep 3;15(9):e0238562. doi: 10.1371/journal.pone.0238562 (PMC7470269; doi:10.1371/journal.pone.0238562)
Supplement: S5 Table — (DOCX) [file pone.0238562.s005.docx]

**S5 Table. Severity factors.**

| **SEVERITY FACTORS** | **Categories** | **ICD-9-CM codes** | **National nomenclature** | **Indicator** |
| --- | --- | --- | --- | --- |
| Metastases | Yes /No | In the index admission: 196, 197, 198 (except 198.81) | - | 605, 606 |
| Histological type | 1. Invasive cancer 2. Carcinoma in situ of breast | 1. 174 2. 233.0 | - | 605, 606 |
| Admission | 1. Hospital 2. Emergency room | - | - | 605, 606 |
| Medical therapy / radiotherapy within 6 months of breast cancer surgery | 1. Medical therapy 2. Radiotherapy 3. Medical therapy and radiotherapy 4. No Medical therapy or radiotherapy | 1. V58.11, V66.2, V67.2   Procedure: 00.10, 99.25, 99.28   1. V58.0, V66.1, V67.1   Procedure: 92.2 | 1. 90.20.5, 99.15, 99.25 2. 38.99.x, 92.21.1, 92.23.x, 92.24.x, 92.25.x, 92.27.x, 92.28.3, 92.28.4, 92.28.5, 92.28.6, 92.29.x, 99.85 | 605, 606 |
