# Supplementary material for: Evaluation of the breast cancer care network within the Lazio Region (Central Italy)
Source: PLoS One. 2020 Sep 3;15(9):e0238562. doi: 10.1371/journal.pone.0238562 (PMC7470269; doi:10.1371/journal.pone.0238562)
Supplement: S6 Table — (DOCX) [file pone.0238562.s006.docx]

**S6 Table. Predictive model.** MBC: Malignant Breast Cancer

| **RISK AND PROTECTIVE FACTORS** | **N** | **RR crude** | **RR ADJ** | **p-value** | **Indicator** |
| --- | --- | --- | --- | --- | --- |
| Age | - | 0.97 | 0.97 | 0.0001 | 605 |
| Medical therapy / radiotherapy within 6 months of MBC surgery | 134 | 0.79 | 0.67 | 0.291 | 605 |
| Admission (emergency room vs hospital) | 19 | 3.37 | 4.47 | 0.011 | 605 |
| Histological type (invasive cancer vs carcinoma in situ of breast) | 3377 | 0.51 | 0.56 | 0.004 | 605 |
| Age | - | 0.89 | 0.88 | 0.0001 | 606 |
| Medical therapy / radiotherapy within 6 months of MBC surgery | 174 | 1.66 | 0.75 | 0.197 | 606 |
| Age | - | 1 | 1 | 0.081 | 608 |
| Age | - | 1 | 1 | 0.749 | 609 |
| Age | - | 0.98 | 0.98 | 0.0001 | 611 |
| Age | - | 0.98 | 0.98 | 0.0001 | 613 |
