# Supplementary material for: Evaluation of the breast cancer care network within the Lazio Region (Central Italy)
Source: PLoS One. 2020 Sep 3;15(9):e0238562. doi: 10.1371/journal.pone.0238562 (PMC7470269; doi:10.1371/journal.pone.0238562)
Supplement: S7 Table — (DOCX) [file pone.0238562.s007.docx]

**S7 Table. Activity volume of the hospitals.**

| **HOSPITAL** | **CITY** | **N** |
| --- | --- | --- |
| LAZIO |  | 6218 |
| A. Gemelli | ROMA | 888 |
| Regina Elena | ROMA | 632 |
| S. Giovanni Addolorata | ROMA | 406 |
| Umberto I | ROMA | 365 |
| S. Andrea | ROMA | 330 |
| Campus Biomedico | ROMA | 315 |
| S. Spirito | ROMA | 278 |
| S. Eugenio | ROMA | 265 |
| S. Camillo | ROMA | 246 |
| S. Pertini | ROMA | 226 |
| C. Fatebenefratelli | ROMA | 215 |
| Tor Vergata | ROMA | 191 |
| S. Maria Goretti | LATINA | 183 |
| S. Filippo Neri | ROMA | 176 |
| SS. Trinità | SORA | 140 |
| S. Giuseppe Marino | MARINO | 117 |
| Belcolle | VITERBO | 112 |
| S. Camillo De Lellis | RIETI | 99 |
| Albano Laziale | ALBANO LAZIALE | 90 |
| S. Paolo | CIVITAVECCHIA | 86 |
| Città Di Roma | ROMA | 76 |
| San Marco | LATINA | 37 |
